# Sex- and Gender-Related Differences in Pruritus in Dermatological Diseases: Insights into Inflammatory, Autoimmune, and Connective Tissue Disorders

**DOI:** 10.3390/life16071182

**Published:** 2026-07-16

**Authors:** Francesca Gorini, Alice Verdelli, Alessandro Magnatta, Simone Landini, Luca Sanna, Rachel Daher, Virginia Corti, Irene Bonanni, Marta Donati, Elena Biancamaria Mariotti, Valentina Ruffo di Calabria, Alberto Corrà, Marzia Caproni

**Affiliations:** 1 Institute of Clinical Physiology, National Research Council, 56124 Pisa, Italy; 2 Rare Skin Diseases Unit, P.O. Piero Palagi, Azienda USL Toscana Centro, European Reference Network-Skin Member, University of Florence, 50122 Florence, Italy; alice.verdelli@uslcentro.toscana.it; 3 Section of Dermatology, Department of Health Sciences, University of Florence, 50125 Florence, Italy; alessandro.magnatta@unifi.it (A.M.); simone.landini@unifi.it (S.L.); luca.sanna@unifi.it (L.S.); rachel.daher@unifi.it (R.D.); virginia.corti@unifi.it (V.C.); valentina.ruffodicalabria@unifi.it (V.R.d.C.); 4 Department of Dermatology, University of Modena and Reggio Emilia, 41124 Modena, Italy; elettradonati@gmail.com; 5 Unit of Dermatology, Azienda USL Toscana Nord Ovest, 55100 Lucca, Italy; elenabiancamaria.mariotti@uslnordovest.toscana.it; 6 Dermatology Unit, Ospedale San Bartolo, 36100 Verona, Italy; alberto.corra.92@gmail.com

**Keywords:** pruritus, chronic itch, sex differences, gender differences, inflammatory skin diseases, autoimmune skin diseases, connective tissue diseases, neuroimmune mechanisms, cytokines, quality of life

## Abstract

Pruritus is a common and burdensome symptom in inflammatory, autoimmune, and connective tissue skin diseases, significantly impairing quality of life, sleep, and psychological well-being. Pruritus arises from a complex interplay between skin barrier dysfunction, immune activation, and neuronal sensitization involving cytokines, alarmins, neuropeptides, and sensory pathways. Increasing evidence indicates that both biological sex and gender-related factors influence itch perception, severity, and clinical expression, although these differences remain insufficiently explored. This review provides a comprehensive analysis of current evidence on sex- and gender-related differences in pruritus across dermatological diseases, with particular attention to the neuroimmune mechanisms underlying chronic itch. Available studies suggest that women more frequently report greater itch intensity, enhanced psychological burden, and higher impairment in daily activities and sleep, whereas men may exhibit different clinical and sensory profiles. However, findings remain heterogeneous because of methodological limitations, small cohorts, and the lack of standardized itch assessment tools. In addition to biological determinants, psychosocial and behavioral factors likely contribute to sex- and gender-specific differences in chronic pruritus. Overall, the available evidence highlights the need for more standardized and sex-informed research approaches to improve the understanding and management of pruritus in dermatological diseases.

## 1. Introduction

Pruritus, or itch, is an unpleasant sensation that triggers an irresistible urge to scratch, and represents the primary, subjective symptom in most dermatological conditions [1,2]. The global prevalence of pruritus is estimated at 39.8%, but this value rises to 56% among patients with skin diseases, particularly those with atopic dermatitis (AD), chronic hand eczema, or psoriasis (PSO) [3]. In addition to being a frequent reason for dermatological consultation, pruritus can substantially impair quality of life (QoL) [2]. Although generally regarded as a benign manifestation, it may persist for several weeks—being defined as chronic when lasting more than 6 weeks—and often becomes highly distressing and difficult to treat [4]. Chronic pruritus (CP), affecting almost one fourth of individuals during their lifetime and being significantly associated with insomnia, depression, and anxiety, is attributable to inflammatory causes in about 60% of patients, with eczema, PSO, and seborrheic dermatitis representing the predominant underlying conditions [5,6]. Pruritus can also arise from endocrine, inflammatory, and metabolic diseases, as well as from neurological and psychiatric disorders, infections, and cancer [2]. Notably, although its exact cause often remains unclear, a wide range of mediators have been identified as key itch-inducing substances [7].

Pruritus, while classically associated with inflammatory skin disorders—an array of conditions characterized by marked activation of both the innate and adaptive immune systems and driven by elevated levels of pro-inflammatory cytokines—is also observed, with varying degrees of severity, in connective tissue diseases [8,9]. These encompass a broad spectrum of disorders, including dermatomyositis, systemic lupus erythematosus, Sjögren’s syndrome, and systemic sclerosis (SSc), all featuring immune dysregulation and multisystem involvement [9].

Furthermore, epidemiological evidence shows that the worldwide prevalence of pruritus is significantly higher in women than in men, with women affected by skin diseases exhibiting markedly higher rates than those without dermatological involvement [3]. Beyond biological sex differences—encompassing anatomical, morphological, physiological, endocrine, and genetic traits—gender-related factors, defined as the sociocultural roles, behaviors, identities, and expectations associated with being female or male, have increasingly attracted attention in medical research, particularly in dermatology. These factors include lifestyle habits, diet, stress exposure, environmental influences, psychological characteristics, and health-related behaviors, all of which may influence the perception and burden of chronic pruritus [10,11,12]. Indeed, females also generally report greater itch intensity and more pronounced psychological impairment, underscoring gender differences in CP that go beyond sex-specific physiological determinants [13].

Such evidence not only illustrates the heterogeneous nature of pruritus but also raises important questions about the contribution of sex-specific biological pathways to its variability across dermatological and autoimmune conditions.

This review aims to provide a comprehensive examination of the current evidence on sex- and gender-related differences in pruritus across inflammatory, autoimmune, and connective tissue diseases affecting the skin, while also addressing the biological mechanisms underlying sex-specific patterns and responses to itch. The review was intentionally restricted to dermatological diseases for which published evidence regarding sex- and/or gender-related differences in pruritus was available. Therefore, it does not represent an exhaustive overview of all pruritic dermatological disorders.

## 2. Literature Search Strategy

A literature search was conducted using the PubMed database to identify studies investigating sex- and gender-related differences in pruritus in inflammatory, autoimmune, and connective tissue diseases affecting the skin. The literature search included articles published up to April 2026. Relevant publications were identified using combinations of keywords related to pruritus, itch, sex differences, gender differences, and the dermatological conditions discussed in this review.

Original research articles, clinical studies, observational studies, and review articles published in English were considered for inclusion. Additional relevant references were identified through manual screening of the reference lists of selected articles.

Studies were included when they provided information on sex- and/or gender-related aspects of pruritus, itch severity, disease burden, pathophysiological mechanisms, or related clinical outcomes. Publications not relevant to the objectives of the review were excluded.

## 3. Pruritus: An Overview

The skin, the largest organ of the human body and accounting for approximately 15% of total body weight, is a highly complex structure that functions as a mechanical barrier, limiting transepidermal water loss while providing protection against infectious agents [8,14]. In contrast, inflammation involving cutaneous structures—whether triggered by external stimuli such as ultraviolet radiation, allergens, irritants, and pathogens, or by internal factors including autoimmune mechanisms or hereditary mutations—can give rise to a wide range of skin disorders [8].

The skin also contains a sophisticated immune compartment, the skin-associated lymphoid tissue (SALT), which comprises various cell types, including Langerhans cells (LCs), keratinocytes, dendritic cells (DCs), macrophages, B and T lymphocytes, and innate lymphoid cells (ILCs) [15,16]. LCs are professional antigen-presenting cells (APCs) that constitute the first line of contact with environmental insults and, after binding foreign antigens, migrate to lymph nodes to initiate adaptive immune responses [17]. Keratinocytes, through their membrane-bound Toll-like receptors, play a central role in the pathophysiology of infectious and inflammatory processes by recognizing conserved pathogen-associated molecular patterns and subsequently activating cascades of innate and adaptive immune responses [15,16]. The dermis, the stromal layer beneath the epidermis, hosts a broad range of immune cell types [17]. Dermal DCs comprise multiple APC subsets specialized in sensing tissue-derived signals [15]. They capture and process antigens within the dermal microenvironment and orchestrate T-cell priming by presenting antigenic peptides via major histocompatibility complex class II (MHC II) molecules [15]. Macrophages, members of the mononuclear phagocytic system, undergo context-dependent phenotypic differentiation and can produce a broad range of cytokines that promote either suppressive (anti-inflammatory) or pro-inflammatory immune responses [15]. B lymphocytes, in addition to playing a key role in humoral immunity through antibody production, also function as APCs capable of activating antigen-specific CD4^+^ and CD8^+^ T cells, while simultaneously exerting important regulatory and cytotoxic activities [18]. The skin further contains distinct populations of T cells—including helper T type 1 (Th1), Th2, Th3, Th17, and regulatory T (Treg) cells—which participate in classical adaptive immune responses and contribute to the maintenance of cutaneous immune homeostasis [15]. Moreover, additional CD4^+^ T-cell subsets, such as Th9, and Th22 cells, have recently emerged as crucial players in skin barrier defense and in the pathogenesis of inflammatory skin diseases [19,20]. Tissue-resident ILCs contribute to the maintenance of barrier integrity and respond to local signals by initiating innate immune pathways, releasing interferon-gamma (IFN-γ) against intracellular pathogens (type 1 immunity) or producing Th2 cytokines in response to environmental triggers (type 2 immunity) [16,21].

Disruption of skin barrier integrity, induced by either exogenous or endogenous pruritic stimuli, elicits an epithelial stress response characterized by the release of multiple pro-inflammatory factors from keratinocytes and resident immune cells [16]. The itch sensation originates from the activation of specialized nerve endings, known as pruriceptors, located at the dermo-epidermal junction and with cell bodies in the dorsal root ganglia (DRG), and is subsequently transmitted through peripheral sensory fibers to the spinal cord, the thalamus, and ultimately to the somatosensory and anterior cingulate cortices [16,22]. CP, in particular, results from a complex, bidirectional interaction between the nervous and immune systems [22]. A wide array of cytokines (e.g., interleukin—IL-4, IL-13, IL-25, IL-31, IL-33, thymic stromal lymphopoietin—TSLP), histamine, serotonin, endothelin, extracellular matrix proteins such as periostin, and proteases (e.g., kallikreins, cathepsins) released by keratinocytes, mast cells, T cells, macrophages, basophils, and eosinophils activate sensory nerve fibers, initiating itch and sustaining a self-perpetuating inflammatory cycle that further amplifies immune activation [16,17,22]. The ensuing inflammatory milieu within the affected skin leads to epidermal thickening and morphological alterations, together with extensive immune-cell infiltration, in which CD4^+^ T cells predominantly display an inflammatory Th2 phenotype [23]. In particular, TSLP, IL-25, and IL-33—epidermis-derived alarmin cytokines—act as key orchestrators of Th2 immunity, promoting activation of Th2 cells, ILC2s, mast cells, and basophils, and their sustained overexpression is strongly associated with chronic allergic inflammation [16,17].

Most pruritic skin diseases are mediated by non-histaminergic neural pathways that activate G-protein-coupled receptors (GPCRs), including protease-activated receptor 2 and 4 (PAR-2 and PAR-4) and Mas-related G-coupled protein receptors C11, A3 and X (MRGPRC11, MRGPRA3 and MRGPRX), expressed on cutaneous sensory terminals [22,24]. Engagement of these receptors ultimately induces the opening of specific ion channels, such as nociceptive transient receptor potential vanilloid 1 (TRPV1) and transient receptor potential ankyrin 1 (TRPA1), followed by membrane depolarization, thereby initiating the molecular events that underlie itch sensory transduction [22,25]. Unlike TRPV1, which is primarily involved in the transduction of histamine-induced pruritus, TRPA1 mediates itch elicited by various non-histaminergic mediators [25]. Nevertheless, the two channels can be co-expressed in the same sensory neurons and act synergistically in both pruriception and nociception, functioning as key transducers of noxious heat stimuli [25]. Notably, among keratinocyte-derived alarmins, TSLP and IL-33 not only serve as master initiators of type 2 inflammation via their effects on a variety of cells including Th2 cells, ILC2s, and basophils, but they can directly stimulate TRPA1-positive sensory neurons, thereby eliciting itch sensation [26,27,28]. Similarly, periostin, after binding to the integrin family cell-surface receptors αVβ3, directly activates itch-transmitting DRG somatosensory neurons [29]. Moreover, periostin release from keratinocytes is induced by TSLP through the JAK/STAT pathway, supporting the existence of a reciprocal TSLP–periostin amplification loop that links the skin to sensory neurons and drives chronic allergic itch [29]. Periostin, together with TSLP or substance P (SP) released by sensory neurons, also induces M2 macrophages to secrete the pruritogenic cytokine IL-31, which directly stimulates pruriceptive nerve fibers and IL-31RA-expressing cells—including macrophages, basophils, and keratinocytes—to amplify inflammation [30,31,32]. IL-4 and IL-13, alone or in combination, act as potent acute pruritogens on nerve endings and exacerbate the Th2 response, further worsening epidermal barrier dysfunction [33,34]. Finally, the proteases kallikrein and cathepsin can elicit itch by activating PAR-2 on keratinocytes or on sensory neurons independently of skin inflammation [35,36], and by directly activating MRGPRs and PAR-2 on sensory neurons, respectively [37,38].

As discussed above, neuropeptide release is triggered by an increase in cytosolic Ca^2+^ concentration following the activation of GPCRs, which in turn engage Ca^2+^-permeable channels, including TRPV1 and TRPA1 [24]. Most cutaneous cells express receptors for neuropeptides—such as SP and calcitonin gene–related peptide (CGRP)—released from sensory nerve endings, particularly C-fibers, thereby establishing a vicious cycle that amplifies the inflammatory response, the so-called neurogenic inflammation [24,39]. SP displays a pronounced pro-inflammatory profile, inducing the production of IL-1α, IL-6, and IL-8 by keratinocytes and enhancing migration and antigen presentation in LCs [24]. Through activation of the cationic molecule receptor MRGPRB2 on mast cells, SP can also directly promote mast-cell degranulation, thereby contributing to the development of allergic skin inflammation [40]. Sensory neurons, acting as primary detectors of allergens, may further activate DCs to migrate to lymph nodes and initiate Th2 differentiation through SP-mediated stimulation of MRGPRA1 [41]. CGRP is a potent microvascular vasodilator involved in the recruitment of inflammatory cells and has been shown to enhance LC antigen presentation in support of Th2 responses, while inhibiting the production of Th1-associated chemokines [24,42]. On the other hand, although generally regarded as a pro-inflammatory mediator, CGRP may also exert anti-inflammatory effects by reducing histamine release [43].

**Figure 1:**
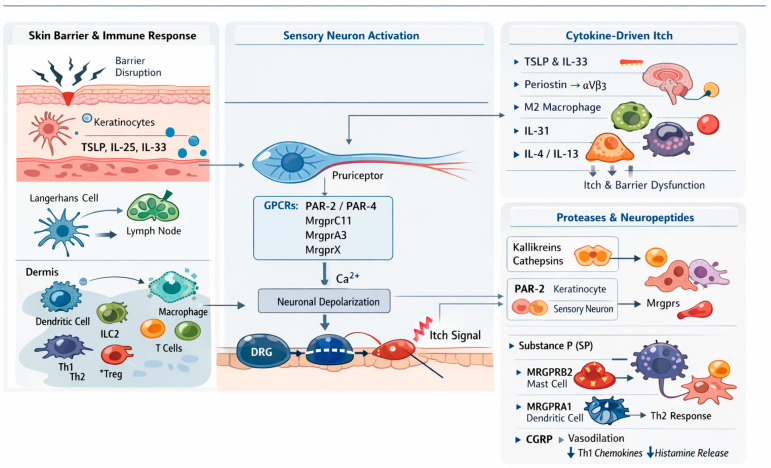
Neuro-immune mechanisms driving chronic itch: Disruption of the skin barrier triggers the release of alarmins (TSLP, IL-25, IL-33) from keratinocytes, activating immune cells within the skin-associated lymphoid tissue. These signals engage pruriceptive sensory neurons via G protein-coupled receptors (PAR-2/4, MRGPRC11, MRGPRC3, MRGPRX), leading to TRPV1/TRPA1 channel opening, Ca^2+^ influx, and neuronal depolarization. Alarmins also induce periostin release, which activates DRG neurons and promotes IL-31 production by M2 macrophages. IL-31, IL-4, and IL-13 amplify inflammation and itch via IL-31RA^+^ cells. Proteases (kallikreins, cathepsins) further activate sensory neurons, while neuropeptides (SP, CGRP) modulate immune responses. SP activates mast cells (MRGPRB2) and dendritic cells (MRGPRA1), while CGRP enhances Th2 responses and inhibits Th1 chemokines. The dermis hosts multiple T cell subsets—including Th1, Th2, Th17, Th22, Th9, Th3, and Treg cells—which orchestrate adaptive immunity and contribute to chronic allergic inflammation. These interactions establish a self-perpetuating neuro-immune loop that sustains pruritus and epidermal dysfunction. Image generated using Microsoft Copilot 365, GPT-5. Abbreviations: CGRP: calcitonin gene–related peptide; GPCR: G protein-coupled receptor; IL: interleukin; ILC: innate lymphoid cell; MRGPR: Mas related G-coupled protein receptor: PAR: protease-activated receptor; TSLP: thymic stromal lymphopoietin; SP: substance P; Th: helper T cell; Treg: regulatory T cell; TRPA1: transient receptor potential ankyrin 1; TRPV1: nociceptive transient receptor potential vanilloid 1.

Thus, the neuro-immune crosstalk underlying the sensory transduction of pruritus is not a unidirectional phenomenon, but rather a dynamic local intercellular network between sensory neurons and peripheral immune cells. Within this network, neurons actively shape the behavior of neighboring cells, thereby fostering an inflamed, pro-pruritic tissue micromilieu (Figure 1).

## 4. Inflammatory Skin Diseases

### 4.1. Atopic Dermatitis

AD is a chronic, complex inflammatory skin disease characterized by recurrent eczematous lesions [44]. It is the leading contributor to the global burden of skin diseases, with approximately 60% of patients developing symptoms within the first years of life, and the highest incidence occurring between 3 and 6 months of age [44]. Males and females are affected differently by AD, with a shift from a male predominance during infancy and childhood to a higher prevalence among females from puberty onward [45]. Recent estimates report a global prevalence of 2.6%, with a higher burden among females compared with males (2.8% vs. 2.4%) [44]. AD represents one of the dermatoses with the highest burden due to pruritus, which typically ranges from moderate to severe intensity and profoundly impairs patients’ QoL, with significant psychosocial consequences and marked interference with daily activities and sleep [46,47,48]. Notably, pruritus in AD appears to correlate with disease severity, yet patients with AD have also been reported to experience severe itch irrespective of clinical stage [48,49]. Additionally, the increased transepidermal water loss characteristic of AD significantly contributes to itch intensity [50]. Although the exact mechanism of pruritus in AD has not been fully established, IL-13 plays a crucial role in AD pathogenesis [51]. IL-13, produced by ILC2s independently of allergen exposure and alarmins, as well as by Th2 cells, acts together with IL-4 to reduce filaggrin expression by interfering with OVOL1 signaling [47]. It also inhibits involucrin expression through an OVOL1-independent mechanism, impairing normal skin barrier function [51]. IL-13 and IL-4, by reducing the production of antimicrobial peptides by keratinocytes, promote skin dysbiosis and subsequent colonization by *Staphylococcus aureus*, contributing to AD development and exacerbation [52]. Both cytokines further amplify Th2-mediated inflammation by promoting Th2 cell differentiation and eosinophil recruitment, as well as enhancing B-cell differentiation and IgE production [51]. The resulting increase in IgE binding to mast cells and basophils leads to enhanced release of histamine and other inflammatory mediators, exacerbating pruritus [51]. Furthermore, the IL-31/IL-33 axis has emerged as a crucial pathway in AD, with IL-33 triggering inflammation and IL-31 amplifying neuronal sensitivity, sustaining a vicious cycle of inflammation, pruritus, and scratching [53]. Finally, histamine through histamine receptors H1 and H4, tryptase, MRGPRX2, and TSLP, have been identified as additional pruritogenic mediators implicated in AD; however, their precise contribution to disease pathogenesis and itch induction remains incompletely understood [54].

Itch is a hallmark of active AD and its most bothersome symptom, often described as the earliest sign of disease relapse, even when the skin does not appear macroscopically affected [55]. Data from the TREATgermany registry—a nationwide, multicenter academic registry in Germany designed for the long-term observation of patients with moderate-to-severe AD and collecting a broad range of physician- and patient-reported outcome measures—indicate that itch was present in 97.2% of the 1134 patients (mean age, 41.0 years, 42.5% female) [55]. Itch severity over the 3 days preceding the baseline visit was evaluated using a numerical rating scale (NRS; 0–10), revealing significantly higher mean intensity in females compared with males (6.1 vs. 5.5; *p* < 0.001) [55]. Consistent with these findings, a Brazilian study evaluating pruritus in 91 patients with AD aged 14–65 years (mean age, 29.7 years, 46.1% female) found that 97.9% of participants experienced pruritus, with a mean NRS intensity of 7.32 over the two weeks preceding baseline [47]. Moreover, higher scores on the Itch-specific Quality of Life Questionnaire (ItchyQoL), a validated 22-item instrument scored on a 1–5 scale, were significantly associated with female sex (*p* = 0.009) [46]. In contrast, an Italian multicenter study [11] recruiting a total of 686 adult patients (age ≥ 18 years, 48.0% female) found no significant sex-related differences in disease severity or duration, itch intensity, or impact on QoL. However, analysis of the relationships among clinical characteristics by multiple correspondence analysis showed that adult males suffer from more severe disease and more intense itch despite appropriate treatment, whereas females with moderate disease severity tend to be undertreated, pointing to a relevant gender-related gap in clinical care [11]. This observed “gender issue” may, at least in part, be driven by the reluctance of women with AD, especially during pregnancy, to use systemic immunosuppressant therapies (i.e., methotrexate, alitretionin, mycophenolate mofetil) due to concerns about fetal safety [56]. The development of emerging biologic therapies (e.g., tralokinumab, lebrikizumab, nemolizumab) for AD targeting inflammatory pathways may open additional treatment opportunities during pregnancy and breastfeeding [56]. However, the limited availability of data from clinical trials—largely due to ethical constraints—currently hampers the ability to draw definitive conclusions regarding their safety and efficacy [56] (Table 1).

### 4.2. Psoriasis

PSO, a common chronic immune-mediated inflammatory skin disease with a global prevalence of approximately 1–3% affecting an estimated 125 million people worldwide, has a complex and multifactorial etiopathogenesis that has not yet been fully elucidated [57,58,59]. Whereas PSO was historically considered a self-limited skin condition, accumulating evidence over recent years has demonstrated that it is a systemic disease associated with multiple extracutaneous manifestations, involving several organ systems and significantly impacting mental health and QoL [59]. Once underestimated, pruritus is now acknowledged as a common and clinically relevant symptom, affecting 70–90% of patients, with baseline itch intensity comparable to that seen in AD among those requiring systemic treatment [57,60]. Recent evidence suggests that pruritus is highly prevalent across different clinical variants of psoriasis, including pustular forms. In a multinational multicenter study evaluating several psoriasis subtypes, including palmoplantar pustular psoriasis and generalized pustular psoriasis, pruritus was reported by 92.9% of patients overall and its prevalence did not significantly differ among clinical variants [61]. Notably, itch severity correlated with disease severity in palmoplantar pustular psoriasis, highlighting the clinical relevance of pruritus also in pustular forms of the disease [61]. Furthermore, pruritus intensity in PSO, beyond its detrimental impact on QoL, including sleep quality, is associated with depressive symptoms and perceived stress [6,62,63]. Although the mechanisms underlying pruritus in PSO remain incompletely understood, a range of mediators has been implicated in itch generation, including neuropeptides such as SP, CGRP, vasoactive intestinal peptide, neuropeptide Y, and somatostatin [63,64,65], suggesting that pruritus in PSO is largely driven by neurogenic inflammation [66]. Stress-induced release of pro-inflammatory neuropeptides from cutaneous nerve endings may further contribute to disease exacerbation [63]. In parallel, serum levels of different chemokines are significantly elevated in PSO patients, with CCL17/TARC being positively associated with pruritus severity as assessed by the 10.0 cm visual analogue scale (VAS) [67]. Among cytokines, IL-31, primarily produced by activated Th2 cells but also by DCs, mast cells, macrophages, eosinophils, and basophils, has been proposed to play a relevant role in PSO pathogenesis, as indicated by its increased levels in both serum and lesional skin of affected patients [62,68,69,70]. However, elevated IL-31 expression does not appear to correlate with itch intensity [62,68,69,70].

Overall, the available evidence on the characterization of pruritus in PSO remains limited, including data on potential sex-related differences. An early study conducted on 109 Swedish patients with a diagnosis of chronic plaque psoriasis (mean age 44.2 years; 35.8% female) reported that females exhibited nearly a four-fold higher pruritus intensity compared with males (odds ratio—OR = 3.8, 95% confidence interval—CI: 2–15; *p* < 0.05) [71]. Based on VAS categorization of pruritus into mild, moderate, and severe, a higher, although not statistically significant, proportion of female patients reported moderate and severe itch (63% and 55% vs. 37% and 45% among men, respectively; *p* = 0.07) [71]. While the authors observed no association between the area of pruritus and sex, differences emerged in itch characteristics, with females reporting stinging, tickling, and crawling sensations in 17%, 16%, and 12% of cases, respectively (vs. 7%, 6%, and 9% in men) [71]. Notably, women had a three-fold higher risk of experiencing tickling compared with men (OR = 3.4; 95%CI: 1–11; *p* < 0.05), and 58% of them reported pruritus as bothersome (vs. 42% of men), although this difference was not statistically significant [71]. Consistently female patients were significantly more likely to receive treatment for pruritus (OR = 3, 95%CI: 1–8, *p* < 0.05) [71]. These findings are in line with those of a previous Italian study enrolling 936 patients with different clinical types of PSO (mean age 45.7 years; 40.7% female), in which, based on Skindex-29 symptom scores (0–5 points), 73.9% of female patients experienced itch often or all the time during the four weeks preceding hospitalization, compared with 56.8% of males (*p* < 0.001), reflecting the sex-related differences reported for all other symptoms [72]. A Turkish cross-sectional study investigating the relationship between pruritus and clinical variable in 880 patients (mean age, 43.8 years; 55% female) found a prevalence of pruritus in psoriatic patients relatively low (62.8%) but significantly associated with female sex [58]. Nonetheless, the presumed higher intensity of pruritus of itch in women should be confirmed after adjusting by age, ethnicity and psychological conditions [71]. A recent Romanian study involving 163 patients (age range 19–87 years, 49.1% female), 70.6% of whom acknowledged experiencing itch, revealed no significant sex-related differences in the proportion of subjects with pruritus based on VAS scores (*p* = 0.06) [64]. However, in the multiple regression model, female patients with greater disease activity (evaluated by the PASI score, 0–10 points) and who more frequently experienced itch episodes had a higher risk of manifesting moderate-to-severe pruritus (OR = 2.63; 95%CI: 1.04–6.66; *p* = 0.04) [64]. This finding corroborates the significant association between itch intensity and frequency (*p* < 0.0001) and suggests that itch perception is influenced by both dimensions [64].

In contrast, none of three Polish studies including 100 (mean age 47.1 years; 40% females), 102 (mean age, 45.2 years; 37.3% female) and 60 patients with PSO (mean age 44.8 years; 25% females) and reporting pruritus in 80.0%, 89.3% and 88.3% of participants, respectively, identified significant sex-related differences in pruritus severity [68,73,74]. Itch intensity was assessed using the VAS (0–10 points) and a 4-Item Itch Questionnaire evaluating distribution, frequency, severity and sleep disturbance (3–19 points), or alternatively the W-AZS-I index, which measures itch extent, frequency and severity together with sleep impairment (0–34 points) [68,73,74]. More recently, Park et al. also did not detect any significant sex-related differences in pruritus intensity, as assessed through the NRS, in a group of 50 Chinese patients (mean age, 45.7 years; 40% female), among whom itch was reported in 80% of cases overall [65] (Table 1).

### 4.3. Prurigo Nodularis

Prurigo nodularis (PN), a long-term inflammatory and intensely pruritic skin disease characterized by hyperkeratotic nodules on the extremities and trunk, is accompanied by frequently intractable chronic itching and repeated scratching, and driven, at least in part, by neural and immune dysregulation [75]. PN is considered a rare disease, with a global prevalence estimated at 0.083%, derived mainly from European and American registry-based studies [76]. On the other hand, PN is frequently underdiagnosed, owing to potential differential diagnoses such as AD, scabies, and other skin diseases with comparable clinical features, as well as psychiatric disorders, given the frequent concurrence of anxiety and depression in patients with PN [75,77]. Furthermore, PN appears to be associated with higher rates of AD, chronic hepatitis C, chronic kidney disease, chronic congestive failure, chronic obstructive pulmonary disease, and type 2 diabetes [78]. While PN was initially described in the early 20th century in a cohort composed exclusively of women, it has since been diagnosed in both sexes, albeit with a slight predominance among female patients [79].

Although the exact pathogenesis of PN remains unclear, with genetic susceptibility and environmental factors likely contributing to disease onset, a persistent “itch-scratch” vicious cycle—driving recurrent excoriation, crusting, and skin thickening—is regarded as the principal mechanism underlying nodule formation [80,81]. The vast majority of patients (>90%) report scratching, and approximately 42% confirm that scratching contributes to itch worsening [82]. In addition to impaired QoL, a considerable proportion of affected individuals is resistant to off-label treatments, given the lack of targeted therapies approved by the US Food and Drug Administration [83]. A broad range of upregulated pruritogens has been detected within PN lesions—including several cytokines (IL-4, IL-6, IL-13, IL-22, IL-31, TSLP, oncostatin M, a member of the IL-6 cytokine family), neuropeptides (SP, CGRP, cortistatin, the latter predominantly secreted by skin mast cells), vasculogenic substances (vascular endothelial growth factor, endothelin-1), nerve growth factor (NGF), and histamine—yet approximately two thirds of patients report itch involving both the nodules and the non-lesional skin [80,84]. In particular, the immune dysregulation in PN is driven by type 2 inflammation, while IL-17 induces endothelin-1 [80,85]. Endothelin-1, in turn, promotes NGF expression in keratinocytes and DRG, and NGF subsequently activates and sensitizes cutaneous C-fibers, fostering the release of SP and CGRP [80,86]. Evidence regarding sex-specific differences in the manifestation of pruritus in PN remains limited. In this context, a large German retrospective study including 1037 patients with CP (mean age, 61.7 years, with men being significantly older than women; 54.8% female), specifically designed to investigate differences between sexes across multiple clinical parameters, including CP-underlying conditions, reported that women more frequently presented with chronic scratch lesions and PN (*p* < 0.001) [13]. In addition, women more often reported sensations of stinging, warmth, and pain, suggesting greater involvement of mechanosensitive C-fibers responsible for the transmission of both pain and itch [13]. Women also obtained significantly higher VAS scores than men at the first visit and more frequently reported that itch was both triggered and modulated by emotional factors [13]. Moreover, in women, CP more often presented in attacks rather than as a continuous symptom, whereas relief and worsening of CP after scratching were not associated with sex differences [13]. Conversely, Whang et al. showed that among U.S. patients with itch (*n* = 18,753; 66.7% female), women were significantly less likely to receive a diagnosis of PN (*p* < 0.001), despite exhibiting a higher prevalence of psychiatric comorbidities compared with men [66] (Table 1).

### 4.4. Lichen

Lichen simplex chronicus (LSC), sometimes referred to as neurodermatitis, is a localized, chronic inflammatory dermatosis characterized by thickened, lichenified plaques that result from a persistent itch–scratch cycle [87,88]. These plaques may become progressively discolored, ranging from pink to dark brown, and may eventually evolve into hypopigmented lesions with a surrounding hyperpigmented border [88]. The localized distribution of LSC, generally involving one or a few anatomical sites—most commonly the neck, ankles, scalp, vulva, pubis, scrotum, and extensor forearms—contrasts with PN, which typically presents with more widespread lesions [87,88,89]. The prevalence of LSC in the general population is estimated at approximately 12%, with adults aged 30–50 years being the most affected; a female predominance has been reported, with a female-to-male ratio of approximately 2:1 [88]. Notably, 20–90% of patients with LSC have a personal or family history of AD, allergic rhinitis, or asthma [88,90]. Consistent with its classification as a psychodermatological disorder, LSC is associated with an increased burden of psychological comorbidities, including stress, anxiety, and depression, alongside reduced neurotrophin levels, and these conditions are related to symptom severity of the LSC [91,92,93]. In turn, the presence of anxiety disorders has been associated with a higher prevalence of LSC [94]. Furthermore, the S/S variant in the polymorphic region of the serotonin transporter gene, which is associated with reduced transcriptional efficiency of the gene promoter and consequently lower serotonin uptake activity, may be related to an increased risk of LSC [95]. Although not life-threatening, severe pruritus in LSC substantially impairs patients’ QoL and frequently disrupts sleep, reflecting its significant psychosocial burden [96,97,98].

In LSC, severe pruritus is the predominant clinical feature, and lesion persistence and progression are driven by repetitive scratching and rubbing [89]. Of note, among pruritic dermatoses, patients with LSC report particularly high levels of scratch pleasurability, with the highest scores recorded in the genital and anal regions, two of the most frequently affected sites [99]. Although the molecular mechanisms underlying pruritus in LSC remain incompletely understood, the condition is predominantly driven by non-histaminergic itch pathways involving pruritogen activation of GPCRs and TRP channels [88]. Moreover, neurotrophins, including NGF, neurotrophin-3 (NT-3), brain-derived neurotrophic factor (BDNF), and glial cell line–derived neurotrophic factor, have been reported to be significantly reduced in patients with LSC compared to healthy controls, although they do not appear to correlate with disease severity [94]. Given the proposed role of NT-3 in anxiety disorders and the negative association between reduced BDNF levels and depressive symptoms in patients with acne vulgaris, together with the bidirectional relationship between LSC and psychological conditions, these findings support the involvement of neuroimmune interactions in the pathophysiology of LSC [88,100,101]. Sex differences in pruritus in LSC have been examined in a limited number of studies. A Spanish study including 103 patients with isolated LSC (mean age. 47.1 years; 68.9% female) and 1184 healthy controls (mean age, 37.6 years; 44.3% female) reported significant sex-related differences in personality traits [91]. Female patients exhibited higher levels of pessimism, emotional reactivity, and suppression of negative emotions compared with both male patients and controls [91]. On the one hand, coping strategies may contribute to emotional distress; on the other hand, stressful situations are known to exacerbate itching and scratching, particularly in women, in whom scratching may represent a maladaptive coping mechanism, suggesting that the intense scratching characteristic of LSC may function as a mild form of self-aggressive behavior [91]. However, the cross-sectional design precludes drawing definitive conclusions regarding the causal relationship between emotional status and scratching in LSC [91]. Furthermore, other factors, including lesion severity, socioeconomic status, and family-related stressors, may also contribute to the initiation or maintenance of scratching behavior [91]. A retrospective analysis including 125 U.S. patients with a primary diagnosis of LSC (mean age 56.6 years; 53% female) found that most patients reported moderate-to-severe pruritus, with NRS scores ranging from 6 to 10 [102]. Patients with multiple lesions exhibited significantly higher mean pruritus intensity compared with those with localized disease (7.8 vs. 7.1 on the NRS; *p* < 0.001) [102]. Psychiatric comorbidities were more prevalent in the multiple-lesion group (50% vs. 26%, *p* = 0.037) [102]. Notably, female patients were more likely to present with multiple lesions (81% vs. 46%; *p* = 0.002), suggesting a higher burden of disease and, consequently, greater itch severity in this subgroup [102] (Table 1).

Taken together, inflammatory skin diseases represent a heterogeneous group of conditions in which pruritus arises from complex interactions between immune activation, neural sensitization, and skin barrier dysfunction. In this context, itch is not merely a consequence of inflammation but reflects dynamic neuroimmune crosstalk and, in many cases, significant psychosocial contributions. Despite shared pathogenic pathways, the expression and intensity of pruritus vary considerably across diseases, highlighting substantial inter-individual and inter-disease variability. Notably, sex and gender emerge as key modifiers of itch perception, severity, and associated burden, likely reflecting both biological differences and psychosocial factors. Overall, this complexity underscores the need for a comprehensive, integrated, and sex-informed framework to better understand and manage chronic pruritus in inflammatory dermatoses.

**Table 1:** Summary table of evidence on pruritus and sex-related differences in inflammatory skin diseases.

Disease	Key Evidence on Pruritus	Reported Sex-Related Differences	Inconsistent Findings— Critical Issues	Knowledge Gaps	References
Atopic dermatitis	Pruritus of moderate-to-severe-intensity in approximately 97–98% of patients; impacting QoL, quality of sleep, and daily activities	German and Brazilian studies: significantly higher itch intensity among females. Italian study: no sex differences in itch intensity	Conflicting results across studies due to heterogenous populations, differences in itch scales (NRS, ItchyQoL), undertreatment of women with moderate disease severity, pregnancy-related treatment avoidance	Lack of longitudinal studies; insufficient data on sex-related differences in treatment responses	[11,46,47,48,55]
Psoriasis	Pruritus affecting 70–90% of patients; comparable to AD among subjects requiring systemic treatment; detrimental effects on QoL; linked to stress, depression, and sleep impairment	Studies conducted in Sweden, Italy, Turkey, and Romania: higher proportion of women reporting higher itch intensity and abnormal, often painful or unpleasant skin sensations: Polish and Chines studies: no sex differences	Studies with small or monocentric cohorts; inconsistent assessment tools (NRS, VAS, Skindex 29 symptom scores, 4 -Item Itch Questionnaire, W AZS I index; limited adjustment for psychological conditions	Lack of longitudinal studies; limited multivariate analyses; poor characterization of sex-specific neuroimmune mechanisms.	[6,57,58,60,62,63,64,65,68,71,72,73,74]
Prurigo nodularis	Severe, chronic, often intractable itch manifesting in more than 90% of patients; involves both lesional and non-lesional skin; strong neuroimmune dysregulation	German study: women present with more scratch lesions and unpleasant skin sensations. U.S. study: women less likely to receive PN diagnosis despite more psychiatric comorbidities	Retrospective design; frequent misdiagnosis in PN; overlap with AD, scabies, psychiatric conditions; age imbalance;	Lack of longitudinal studies; limited data on differential therapeutic response; poor understanding of sex-related sensory profiles.	[13,66,80,81,82,83,84]
Lichen simplex chronicus	Chronic, often severe pruritus driven by a persistent itch–scratch cycle; substantial impact on QoL and sleep; high “scratch pleasurability” reported	Women more frequently exhibit psychological comorbidities and multiple-lesion disease, associated with higher itch severity	Limited number of studies; mostly cross-sectional or retrospective; lack of standardized itch assessment; difficulty disentangling psychological vs. biological drivers	Need for longitudinal studies; limited understanding of sex-specific neuroimmune and psychodermatological mechanisms; unclear causal relationship between psychological factors and scratching behavior	[89,91,96,97,98,99,102]

Abbreviations: AD: atopic dermatitis; ItchyQoL: Itch-specific Quality of Life Questionnaire; NRS: numerical rating scale; QoL: quality of life; VAS: visual analogue scale.

## 5. Autoimmune Bullous Diseases

### 5.1. Bullous Pemphigoid

Bullous pemphigoid (BP), the most common pemphigoid disorder, is a chronic autoimmune blistering disease of the skin and mucous membranes, driven by eosinophilic inflammation and autoantibodies targeting the hemidesmosomal proteins BP180 and BP230 [103,104]. BP predominantly affects older individuals, and advancing age, along with recognized risk factors such as neurological disorders including dementia, epilepsy, Parkinson’s disease, and stroke, may partly explain the increasing annual incidence of the disease, currently estimated at 8.2 per 100,000 people worldwide [104,105]. A clear female predominance has been observed in most studies, with a female-to-male ratio ranging from 1.04 to 5.1 [106]. BP is also a potentially fatal disease, with reported worldwide mortality rates varying markedly—from 27% to 72%—and is frequently accompanied by multiple comorbidities, including hypertension, diabetes mellitus, and neuropsychological conditions [103]. In a preclinical stage, characterized by the absence of skin lesions in approximately 20% of patients, pruritus may represent the sole presenting symptom [107,108]. Conversely, severe pruritus is a hallmark of BP and substantially impairs patients’ QoL, while therapeutic options specifically targeting itch remain limited due to an incomplete understanding of its underlying pathophysiology [109]. In this context, eosinophils within BP blister fluid have been identified as a major source of the pruritogenic cytokine IL-31, linking eosinophilic inflammation to BP-associated itch [110]. Although high levels of histamine have been detected in blister fluids, together with mast-cell infiltration in early BP lesions, mast-cell–derived histamine does not appear to be a central contributor to BP-associated pruritus, whereas histamine released from basophils may represent a more relevant driver [111,112].

To date, four studies have examined sex differences in pruritus prevalence or severity. In a Polish cohort of 28 patients with BP (mean age, 74.5 years; 75% female), no significant sex-related differences in pruritus intensity were observed using the 4-Item Itch Questionnaire: women reported a mean score of 11.4 points, and men a mean score of 10.4 points [113]. Consistently, no significant differences were found using the NRS, which showed moderate-to-severe itch intensity in both sexes [113]. A multicenter French study enrolling 60 patients with BP (mean age, 77.4 years; 50% female) reported that 85% of subjects experienced daily pruritus, with moderate intensity in 43% of cases and 60% reporting itch for more than 6 h per day, based on the 5-D Itch Scale [107]. In the multivariate linear regression used to identify factors associated with pruritus severity in BP, sex was not significantly associated with symptom intensity, in line with the lack of association between sex and overall disease severity [107]. In a retrospective cohort study from the United States including patients diagnosed with autoimmune blistering diseases—31 of whom were affected by BP (mean age, 70.3 years, 74.2% female)—sex did not significantly influence itch-related QoL [114]. The study conducted by Mazan et al. in a cohort of 55 Polish patients with BP (mean age, 79 years; 60% female) showed that although pruritus was more frequently observed in women than in men (73.3% vs. 55.6%), this difference did not reach statistical significance [115]. Similarly, the authors also found no significant sex-related differences in the morphology of skin lesions, nor in the treatments administered [115]. Given the relatively higher incidence of BP among females at the global level [116], inaccuracies in patients’ medical histories—including those related to symptom reporting—cannot be excluded [115]. Furthermore, the authors noted that medical records did not include information on pruritus preceding the onset of characteristic skin lesions [115] (Table 2).

### 5.2. Pemphigus

Pemphigus comprises an epidemiologically heterogeneous group of autoimmune bullous diseases, among which pemphigus vulgaris (PV), accounting for approximately 70% of cases, and pemphigus foliaceus (PF), representing about 20%, are the two major subtypes [104,117,118]. Although the introduction of systemic corticosteroids has markedly reduced mortality—particularly in PV—pemphigus remains a potentially life-threatening condition, with mortality rates estimated between 5–9% [108,117,118]. PV, driven by autoantibodies directed against the desmosomal proteins desmoglein 1 and desmoglein 3, which are essential for keratinocyte cohesion, is characterized by flaccid blisters and erosions involving the skin and mucous membranes [104,119]. Although PV may occur at any age, it most frequently presents between the fourth and sixth decades of life and is slightly more common in females [120]. Reported incidence rates vary widely, from 0.1 to 5 per 100,000 person-years, reflecting substantial geographic and ethnic differences [119]. Sporadic PF is an infrequent disease, with an estimated incidence of fewer than 1 case per million people, while higher prevalence rates have been reported until the early 2000s in some rural regions of Africa and South America, potentially linked to poor hygiene and housing conditions [117]. In contrast to PV, PF involves individuals across a broader age spectrum and is defined by subcorneal acantholysis, which leads to the formation of superficial and easily ruptured flaccid blisters [121]. PV has traditionally been regarded as a Th2-driven disease [122,123]. However, more recent evidence indicates a broader immune dysregulation, with elevated levels of Th2 cytokines (IL-4 and IL-5) alongside Th17-related cytokines, including IL-22 and IL-23, compared with healthy controls [122].

Evidence on pruritus in pemphigus remains scarce; however, available data suggest that approximately half of patients with PV experience itch [124]. Two studies have investigated whether sex influences pruritus-related outcomes in patients with pemphigus. In a cohort of 36 American individuals diagnosed with PV or PF (mean age, 54.2 years; 58.3% female), Cole et al. found that ItchyQoL scores were significantly higher in patients with PF compared with those with PV (*p* = 0.04) but were not correlated with either disease duration or sex [114]. In a U.S. study including 81 patients with pemphigus (65.5% PV; 65.5% female), itch intensity was assessed using the pruritus component of the Bullous Pemphigoid Disease Area Index, which rates itch severity on a 0–10 scale over the preceding day, week, and month, as the Pemphigus Disease Area Index does not include a specific itch assessment [125]. In line with the findings reported in [114], a diagnosis of PF was associated with greater itch intensity compared with PV (*p* = 0.007), whereas neither, disease severity, ethnicity nor sex showed a significant association with itch severity [125] (Table 2).

### 5.3. Dermatitis Herpetiformis

Dermatitis herpetiformis (DH), the relapsing cutaneous manifestation of celiac disease (CD), is characterized by severely pruritic grouped erythematous papules or plaques symmetrically distributed on the scalp, nuchal area, elbows, knees, and buttocks [126,127,128]. A further hallmark of DH is the presence of granular IgA deposits within the dermal papillae and/or along the dermoepidermal junction [126]. DH is a rare disease occurring predominantly among Caucasian individuals, with a reported prevalence between 11.2 to 75.3 per 100,000 people and incidence between 0.4 to 3.5 per 100,000 per year [127,128]. While the highest disease prevalence rates have been recorded in Finland (75.3 per 100,000 people), DH is extremely rare among African and Asian populations owing to the absence of the human leukocyte antigen haplotypes DQ2 and DQ8—consistently found in Caucasian patients with DH and strongly associated with CD—and to the low wheat consumption in these geographic areas [128,129,130]. Over the last decades, despite the increasing incidence of CD, the incidence of DH has shown an opposite trend [126,128]. This divergence is thought to reflect the greater awareness of both physicians and patients regarding CD and the consequent rise in CD screening tests, which has led to the identification of latent or potential CD cases [127]. Although DH can occur at any age, its presentation peaks between 30 and 40 years and, unlike CD, it shows a male predominance, with a male-to-female ratio ranging from 1.5:1 to 2:1 [110]. More recent studies, however, have not confirmed substantial sex-related differences in prevalence [131].

In DH, pruritus is the leading symptom and may even precede the appearance of skin lesions by several months [128,132]. The pathogenesis of pruritus in DH, however, is not fully understood; neurogenic inflammation, mechanical itch dysesthesias, and the release of inflammatory cytokines are all thought to contribute [128]. IL-31, in particular, has attracted considerable recent interest because of its potential contribution to itch in inflammatory skin diseases (see previous sections). Serum levels of IL-17 have been reported to be significantly higher in patients with DH than in healthy controls, together with increased expression of IL-31 in infiltrating cells within skin lesions [133]. Conversely, another study reported significantly lower serum IL-31 concentrations in patients with DH compared with controls [113]. This finding may reflect mast-cell hyperactivation: in addition to promoting degranulation, activated mast cells may upregulate IL-31 receptor mRNA expression, thereby increasing receptor availability and contributing to the reduced circulating levels of IL-31 [113]. Available data on pruritus expression in DH show limited characterization of potential sex-related differences between male and female patients. In their assessment of itch intensity in 24 Polish patients with DH (mean age 41.9 years; 58.3% female), Kulczycka-Siennicka et al. found that 20.8% of patients with DH reported the maximum score on the NRS; however, no significant sex-related differences were identified using the 4-Item Itch Questionnaire [113]. In a cohort of 237 long-term–treated patients with DH (median disease duration 24 years; 46.8% female), no significant sex-related differences were observed in the frequency of DH-related skin symptoms, despite women showing better adherence to a gluten-free diet (*p* = 0.022), the only effective treatment for DH [134,135]. Consistently, no significant differences were detected in gastrointestinal symptoms between sexes [134]. Notably, the gluten-free diet appears to exacerbate gender differences in meal planning, grocery shopping, and cooking, owing to its complexity and time-intensive demands, with the burden falling disproportionately on women [136]. However, only 4% of female patients regularly used dapsone—a sulfone drug with potent anti-inflammatory properties that relieves itch and rash in DH within a few days—compared with 13% of male patients [131,134] (Table 2).

Collectively, pruritus in autoimmune bullous diseases reflects immune-mediated mechanisms driven by autoantibody-induced inflammation and complex cellular interactions within the skin. Although itch has traditionally been considered a secondary feature in these conditions, growing evidence highlights its clinical relevance and impact on patient’s QoL. However, the available data remain limited and heterogeneous, and the underlying mechanisms of pruritus are still incompletely defined. In particular, evidence regarding the role of sex and gender in modulating itch in autoimmune bullous diseases is scarce and inconsistent. This lack of robust and standardized data emphasizes the urgency for further research to clarify the pathophysiology of pruritus and to better characterize its clinical burden, including potential sex-related differences.

**Table 2:** Summary table of evidence on pruritus and sex-related differences in autoimmune bullous diseases.

Disease	Key Evidence on Pruritus	Reported Sex-Related Differences	Inconsistent Findings— Critical Issues	Knowledge Gaps	References
Bullous pemphigoid	Severe pruritus is a hallmark; may be the only symptom in ~20% of patients;	Across four studies (conducted in Poland, France, and United States) no significant sex-related differences in itch intensity, NRS scores, 4-Item Itch Questionnaire, or itch-related QoL. A Polish study showed higher pruritus frequency in women (73.3% vs. 55.6%), but not statistically significant.	Higher global incidence in females but no sex differences in itch severity; small cohorts; retrospective design; possible inaccuracies in symptom reporting; heterogeneous itch scales (NRS, 4-Item Itch Questionnaire, 5-D Itch Scale)	Lack of prospective studies; no studies on sex-related differences in response to antipruritic treatments.	[107,108,109,113,114,115]
Pemphigus	Pruritus is understudied; ~50% of PV patients report itch; PF consistently associated with greater itch intensity than PV	Two U.S. studies found no significant association between sex and itch severity or itch-related QoL	Very limited number of studies; small sample sizes; itch not included in standard PV scoring system; BPDAI pruritus component for pemphigus.	No exploration of sex-specific sensory or inflammatory pathways; insufficient data on sex-related differences in itch burden or treatment response.	[114,124,125]
Dermatitis herpetiformis	Pruritus is the leading symptom and may precede lesions by months	No significant sex-related differences in itch intensity (NRS, 4-Item Itch Questionnaire) or frequency of skin symptoms. Women show better adherence to gluten-free diet but use dapsone less frequently (4% vs. 13%).	Lack of standardized itch assessment; potential confounding from dietary adherence; unclear whether sex influences cytokine profiles or pruritus pathways.	No data on sex differences in response to gluten-free diet or dapsone regarding itch; poor understanding of sensory phenotypes by sex.	[113,128,132,134]

Abbreviations: BPDAI: bullous pemphigoid disease area index; NRS: numerical rating scale; PF: pemphigus foliaceus; PV: pemphigus vulgaris; QoL: quality of life.

## 6. Organ-Specific Autoimmune Skin Diseases

### 6.1. Lichen Sclerosus

Lichen sclerosus (LS) is a chronic, lymphocyte-mediated disorder that predominantly affects mucocutaneous tissues, with a marked predilection for anogenital sites, although extragenital involvement in neck, shoulders, upper trunk, thighs, and oral cavity may also occur in a minority of patients [137,138]. At disease onset, symptoms are often subtle and may vary considerably between patients [139]. Early manifestations include itch and burning, and in some cases pain, as well as white patches or depigmentation of the genital skin [139,140]. These features may progressively evolve into erythema, fissures, and scarring, ultimately leading to urinary and sexual dysfunction; such chronic changes are also associated with an increased risk of genital malignancy [137,139,140,141]. The etiopathogenesis of LS remains incompletely understood; however, immune dysregulation is thought to play a central role in disease pathogenesis and progression [142]. At the molecular level, LS is characterized by the upregulation of pro-inflammatory cytokines (e.g., IL-1, IL-7, IL-15, IFN-γ, and TNF-α), reflecting a predominantly Th1-skewed immune response, together with the presence of circulating IgG autoantibodies targeting extracellular matrix protein 1 (ECM1) and antigens (e.g., BP180, BP230) of the basement membrane zone [138,142,143]. ECM1 autoantibodies are thought to inhibit matrix metalloproteinase-9 activity, resulting in increased dermal collagen synthesis—especially of types I and III—and promoting the development of a sclerotic phenotype [138,144]. Furthermore, although genetic susceptibility in LS has been linked to genes involved in HLA class II antigen regulation, familial cases are relatively uncommon (approximately 12%), and the exact mode of inheritance has yet to be fully elucidated [141,145]. Although LS can occur at any age and in both sexes, it shows a marked female predominance, with a reported female-to-male ratio ranging from 6:1 to 10:1 [137,146]. It also presents a bimodal age distribution. In females, vulvar LS is characterized by a first peak in prepubertal girls and a second peak during the peri- and postmenopausal period [137,138]. Males exhibit a comparable age-related distribution, with a first peak after puberty, typically in the third decade of life, and a second peak after 60 years of age [138]. The exact prevalence of LS remains uncertain, likely due to frequent misdiagnosis, underdiagnosis, and the absence of symptoms in up to one-third of cases [138]. Despite these limitations, available data suggest that LS is a relatively rare condition, with estimated prevalence ranging from 0.1% to 0.3% [147].

An Italian multicenter cross-sectional study including 729 patients with genital LS (mean age, 57.3 years; 53.8% female) reported a high prevalence of pruritus, affecting 62.3% of patients, followed by burning (56.2%) and dyspareunia (40.6%) [148]. Notably, all three symptoms were more frequently observed in females than in males (*p* < 0.001), with women also exhibiting greater symptom severity (*p* < 0.001) [148]. As a result of more severe itch, hyperkeratosis, ecchymosis, and itch-related excoriations, were significantly more frequent in females than in males [148]. Of note, a correct referral diagnosis was more common among female patients (*p* = 0.03), who were more frequently referred by gynecologists, whereas male patients were more often referred by general practitioners [148]. Furthermore, a greater proportion of female patients had received treatment before study inclusion, with significantly higher use of moisturizers and emollients compared with males [148]. Therefore, although evidence is currently limited to a single study, LS appears to be more frequent and severe in females than in males, with women also experiencing a more pronounced negative impact of the disease on sexual function (Table 3).

### 6.2. Vitiligo

Vitiligo is a common acquired skin disorder characterized by the loss of melanocytes, resulting in well-defined depigmented macules and patches [149,150,151]. It is regarded as a chronic autoimmune disease with a complex and multifactorial etiology [152,153]. Although numerous pathogenic mechanisms have been proposed—including genetic susceptibility, cytotoxic immune responses, the presence of anti-melanocyte antibodies, defects in melanocyte adhesion, and biochemical and neurogenic damage—the pathogenesis of vitiligo has not yet been fully understood [108,150,153]. However, vitiligo is now recognized as more than a purely cutaneous disorder, being associated with a range of organ-specific and systemic conditions, including ocular and auditory abnormalities, neurological and psychological disorders, as well as endocrinological and cardiometabolic diseases [150]. The prevalence of vitiligo ranges from 0.1% to 2% globally, but may reach approximately 2.5% in certain populations, particularly in Africa [154]. Given its visible cosmetic impact and the absence of curative treatment, vitiligo is associated with substantial psychological burden, including low self-esteem and impaired QoL, with female patients reporting significantly worse scores than male patients [155]. Depression and anxiety are the most commonly reported psychosocial comorbidities in vitiligo, affecting approximately 35% of patients, particularly women, and contributing significantly to the overall disease burden [156,157].

Despite its generally asymptomatic nature, itch can affect approximately 20% of patients and may precede the onset of cutaneous lesions [149,152,153]. The pathogenesis of pruritus in vitiligo has yet to be fully elucidated. Nonetheless, similarly to many dermatological diseases, itch is thought to arise from neurogenic inflammation, with mediators such as neuropeptides released from dermal nerve endings following various stimuli [153]. Additionally, IL-31, a key mediator of pruritus in multiple chronic skin disorders, is elevated in patients with vitiligo, with significantly higher levels observed in those experiencing pruritus compared with healthy controls [152]. Notably, IL-31 levels correlate positively with disease severity, as assessed by the Vitiligo Area Scoring Index and Vitiligo Disease Activity score, as well as with itch intensity, measured using the 5-D Itch Scale, suggesting that IL-31 may serve as a reliable biomarker of disease activity in vitiligo, beyond its association with pruritus [152]. In evaluating sex-related differences in pruritus presence among Egyptian patients with vitiligo, Lashin et al. found no significant differences between males and females [152]. A Thai study including 402 participants (mean age, 45.4 years; 64.2% female) reported a pruritus prevalence of 20.2%, with a median intensity of 5 on the VAS (1–10) [153]. Severe pruritus was documented in nearly one-third of patients (28.4%) and showed a trend toward higher intensity in females compared with males, although this difference did not reach statistical significance (33.3% vs. 18.6%; *p* = 0.291) [153]. Conversely, crawling sensations were more frequently reported by women than by men, with a trend toward statistical significance (*p* = 0.069) [153]. Furthermore, pruritus was associated with impairment in daily activities and sleep disturbance in 60.5% and 39.5% of patients, respectively; notably, women were disproportionately affected among patients reporting moderate-to-severe activity impairment (*p* = 0.033) (Table 3).

Overall, organ-specific autoimmune skin diseases, including LS and vitiligo, represent a distinct group of disorders characterized by targeted immune-mediated injury to specific cutaneous components. Although pruritus has traditionally been underestimated in these conditions, emerging evidence indicates that it may contribute to disease burden in a significant subset of patients. The pathophysiology of itch in these disorders likely involves a complex interplay between immune activation and neuroimmune signaling pathways, including cytokine-mediated mechanisms. However, the current evidence is limited and often heterogeneous, particularly with respect to sex- and gender-related differences. These factors, while recognized as important modifiers of disease expression and psychological burden, remain poorly characterized in the context of pruritus. Further research is therefore warranted to elucidate the biological and clinical determinants of itch in organ-specific autoimmune skin diseases.

**Table 3:** Summary table of evidence on pruritus and sex-related differences in organ-specific autoimmune skin diseases.

Disease	Key Evidence on Pruritus	Reported Sex-Related Differences	Inconsistent Findings— Critical Issues	Knowledge Gaps	References
Lichen sclerosus	Pruritus is a common and often prominent symptom (up to ~60% of patients); associated with burning and dyspareunia; may be associated with impairment of daily activities, sleep disturbance, and sexual dysfunction	Higher symptom severity and burden in women; more frequent and severe itch-related impairment, including sexual dysfunction	Limited number of studies; cross-sectional data; overlap with pain and burning sensations	Limited data on pathophysiological mechanisms of pruritus; lack of longitudinal studies; unclear relationship between immune-mediated damage, fibrosis, and itch	[140,148]
Vitiligo	Pruritus reported in approximately 20% of patients and may precede lesion onset; associated with impaired QoL	Higher psychological burden and QoL impairment in females; no consistent sex differences in pruritus prevalence or intensity across studies	Limited number of studies specifically evaluating pruritus; heterogeneous methodologies; variability in itch assessment tools; some findings do not reach statistical significance	Limited understanding of mechanisms underlying pruritus; insufficient data on sex- and gender-related differences in itch	[149,152,153]

Abbreviations: QoL: quality of life.

## 7. Connective Tissue Diseases

### 7.1. Systemic Sclerosis

Systemic sclerosis (SSc), a chronic and complex immune-mediated connective tissue disease also known as scleroderma, result from a combination by vascular damage and progressive tissue fibrosis affecting the skin and multiple internal organs, and is associated with high morbidity and mortality [158,159,160]. Clinical manifestations range from Raynaud’s phenomenon and fatigue to severe complications such as interstitial lung disease, pulmonary arterial hypertension, esophageal dysmotility leading to heartburn and/or dysphagia, and, more rarely but life-threateningly, scleroderma renal crisis [158,160]. SSc has traditionally been considered a rare disease, with recent estimates indicating a global prevalence of 18.87 per 100,000 people and approximately 0.67 million new cases diagnosed annually worldwide [158,159]. Although SSc may occur at any age, it predominantly affects adult women, a pattern shared with several other autoimmune diseases [159]. The disease course of SSc is often rapidly progressive and leads to substantial impairment of QoL [159]. Furthermore, given the heterogeneous presentation of SSc and the evolving diagnostic and classification criteria, patients may face difficulties in accessing appropriate healthcare, and diagnosis is often challenging for clinicians in the early stages of the disease [159,161]. Skin involvement in SSc shows marked variability both between patients and within the same patient over time, with skin fibrosis and Raynaud’s phenomenon representing the most common manifestations (>90% of cases), followed by telangiectasia, digital ulcers, and calcinosis [161,162].

Pruritus affects approximately 40–65% of patients with SSc, with frequent onset during the early stages of the disease, but tends to decline as the disease progresses [108,163]. Of interest, pruritus involves not only sclerotic areas but also non-affected skin sites, occurring in 61% of patients [164]. The pathophysiology of pruritus in SSc remains largely unknown; nevertheless, a wide range of mediators has been proposed as potential pruritogens in this disease [162]. Lysophosphatidic acid (LPA), which is generated at sites of inflammation or cell injury, may play an important role in the pathogenesis of SSc [165]. Autotaxin (ATX), whose levels are increased in the fibrotic skin of patients with diffuse SSc, participates in the ATX–LPA–IL-6 axis involved in the development and progression of SSc fibrosis [166]. Moreover, ATX levels have been shown to positively correlate with pruritus intensity [167]. Increasing evidence has implicated Toll-like receptor (TLR) signaling—which plays a central role in innate immunity against pathogens—as a potential contributor to SSc pathogenesis [168]. Moreover, TLR expression in primary sensory neurons, including dorsal root ganglia and trigeminal ganglion neurons, has been linked to the modulation of pain and itch sensations [169]. Notably, although Th22 cells appear to contribute to the development of skin fibrosis in patients with SSc, the disease is generally regarded as Th2-dominant, and type 2 cytokines such as IL-4 and IL-13 can directly activate pruriceptive neuronal pathways in both mice and humans, eliciting responses comparable to those induced by canonical pruritogens like IL-31 [26,170]. Only a limited number of studies has evaluated whether the manifestation of pruritus in patients with SSc may be influenced by sex. In a Canadian cohort of 400 patients with SSc (mean age 56.3 years; 87.8% female), 44.8% reported pruritus on most days, and the presence of pruritus was independently associated with the number of gastrointestinal symptoms [163]. Women accounted for 87.2% of the subgroup with pruritus (156 of 179), but the prevalence of pruritus did not differ significantly between sexes [163]. In a subsequent analysis based on an expanded sample from the same registry, 959 patients (mean age, 56.3 years; 87.2% female) were assessed at two consecutive annual visits and asked whether they had experienced pruritus on most days in the past month, using the same dichotomous yes/no item applied in the original study [171]. Pruritus had a prevalence of 42.6% in this cohort, and 86.8% of patients reporting pruritus were women; however, no significant associations were found with sex or disease duration [171]. It should be noted that reliance on self-report and the use of a dichotomous item to assess pruritus may introduce potential bias. Although the proportion of patients reporting pruritus does not appear to change substantially over time, symptom severity may fluctuate, and such variations cannot be captured with this type of assessment [171]. In a French study, 82 patients with SSc (mean age, 63 years; 82.5% female) were invited to retrospectively complete a standardized questionnaire assessing the intensity, chronology, location, and qualitative characteristics of pruritus, as well as other associated sensations [162]. Pruritus severity was additionally evaluated using the 5-D Itch Scale, analysis [162]. Consistent with previous findings, the comparison between patients with and without pruritus revealed no significant associations with sex, age, disease duration, SSc subtype, or organ involvement, including skin involvement [162] (Table 4).

### 7.2. Morphea

Morphea, also known as localized scleroderma, is a rare connective tissue disease with a still incompletely understood etiopathogenesis [172]. It is characterized by an initial inflammatory phase with erythematous patches on the hands, neck, trunk, and extremities, followed by the development of sclerotic dermal changes and subsequent atrophy [92,173]. Based on the extent and depth of fibrosis, morphea is classified into five main subtypes, including circumscribed, generalized, linear, deep, and mixed variants, and diverse subtypes which may also be associated with extracutaneous manifestations, including musculo-articular, neurological, and ocular disorders [173,174]. The annual incidence of morphea is estimated to range from 4 to 27 new cases per million people, with a female-to-male ratio of 2–4.2:1 [173,174]. Incidence is similar in adults and children and shows two recognized peaks: the first occurring around a mean age of 10 years, and the second during the fifth decade of life [172,173]. Although the cause of morphea is unknown, several triggers—including infections, local trauma, vaccinations, and surgical procedures—may promote immune system activation, leading to excessive collagen deposition, consequent fibrosis, and endothelial and vascular injury [175].

To date, information on the prevalence and patterns of pruritus in morphea is sparse, and no studies have specifically examined sex differences in its occurrence or severity. Nonetheless, given the higher overall prevalence of morphea in females, it is plausible that pruritus may also be more frequently reported by women. Symptoms such as pain and itch are strongly associated with poor health-related quality of life (HRQoL) [176,177]. Furthermore, a cross-sectional study including 110 Hungarian patients (mean age 56.8 years; 84% female) identified sex, along with disease severity, disease subtype, use of systemic therapy, and involvement of the hands and/or feet, as determinants negatively affecting the Dermatology Life Quality Index (DLQI) (scores 0–30, with higher scores indicating poorer HRQoL) [178]. Female sex, in particular, was associated with significantly higher DLQI scores compared with males, and itchy or painful skin (46%) was among the most frequently reported issues [178]. These findings are consistent with a previous U.S. cross-sectional study of 233 adults (mean age 37.4 years; 84.1% female), in which itch was the most frequently reported symptom and, similarly to female sex, was associated with poorer QoL [177]. Furthermore, in a Polish cross-sectional study involving 40 patients with morphea (median age, 46.5 years; 82.5% female), female sex was the only demographic or clinical factor that negatively affected scores on the emotion subscale of the Skindex-29, a validated, skin-specific instrument comprising 29 items to assess HRQoL [179]. In addition, symptoms such as pruritus were strongly associated with lower scores in the physical component of the Short Form-36 (SF-36) global health-related quality-of-life measure [179]. Morphea, which can lead to permanent damage of the skin and subcutaneous tissue, negatively affects aesthetics and self-esteem and, consequently, the psychological well-being of patients, particularly women [179]. Taken together, these findings suggest that pruritus in female patients with morphea may be more common and may contribute to impaired HRQoL (Table 4).

### 7.3. Dermatomyositis

Dermatomyositis (DM) is an autoimmune inflammatory myopathy characterized by immune-mediated muscle weakness and distinctive cutaneous manifestations, such as Gottron’s papules, heliotrope rash, and facial erythema which may precede, follow shortly after, or occur concomitantly with muscle involvement [92,180,181]. Reported estimates indicate an incidence of 1.1 per 100,000 person-years and a prevalence of 13 per 100,000 for DM, with a bimodal age distribution (juvenile onset and mid-to-late adulthood [182,183]. Although the etiology of DM remains unknown, established risk factors include female sex, genetic susceptibility, exposure to ionizing radiation, a history of respiratory diseases, and various environmental factors related to geographic location [182]. Despite being relatively uncommon, DM is associated with reduced life expectancy, with a 2.4–7.5-fold higher mortality rate compared to matched controls, largely due to its link with interstitial lung disease, cardiovascular complications, and malignancies [182]. Furthermore, patients with DM exhibit poorer QoL across all SF-36 subscales compared to the general population, with particularly reduced scores in vitality and mental health relative to those observed in several other chronic dermatological and non-dermatological diseases [184].

Pruritus is a commonly reported symptom in patients with DM, with a prevalence ranging from less than 50% to more than 90%, and is significantly associated with poorer QoL, although it does not substantially affect the emotional component [184,185,186]. The severity of pruritus in patients with DM is often moderate to severe, as measured by the VAS [186,187] and correlates with increased cutaneous disease severity [186]. The mechanisms underlying pruritus in DM remain to be fully elucidated; however, Kim et al. [186] showed that the expression of IL-31 and its receptor was upregulated in lesional skin compared with non-lesional skin and healthy controls. As previously reported, IL-31—primarily produced by activated CD4+ T cells, in particular activated Th2 cells, mast cells, macrophages and dendritic cells—plays a central role in cutaneous innate and adaptive immunity and is upregulated at both the transcriptional and protein levels in pruritic dermatoses [188]. Consistently, IL-31 mRNA expression positively correlates with VAS itch scores in DM [186]. A case study also suggested that scalp pruritus in DM may be attributable to a reduced density of epidermal nerve fibers and structural abnormalities in the remaining fibers, despite minimal differences between patients with DM and healthy controls in CGRP and SP expression within the subepidermal neural plexus [189]. To date, a single study has assessed sex differences in pruritus in DM. In a cohort of 191 U.S. patients (mean age, 50.9 years; 85.3% female), pruritus was reported by most individuals, with only 9.4% remaining asymptomatic [186]. Female patients tended to report greater itch severity than males (median VAS: 4.0 vs. 1.9, respectively; *p* = 0.52), with moderate-to-severe pruritus observed in 52.1% of females compared with 42.9% of males; however, this difference did not reach statistical significance [186]. This trend is nonetheless consistent with patterns observed in other pruritic skin diseases (Table 4).

In sum, pruritus in connective tissue diseases arises within a distinct pathophysiological framework characterized by the interplay of immune dysregulation, fibrotic processes, vascular dysfunction, and alterations in cutaneous innervation. In this setting, itch cannot be solely attributed to inflammation but reflects complex structural and neuroimmune changes within the skin. While data on sex and gender differences remain limited, these factors may contribute to variability in itch perception and clinical burden, highlighting the need for dedicated studies in this area. Compared with inflammatory dermatoses, pruritus in connective tissue diseases appears less consistently characterized, with a greater contribution of structural and fibrotic mechanisms.

**Table 4:** Summary table of evidence on pruritus and sex-related differences in connective tissue diseases.

Disease	Key Evidence on Pruritus	Reported Sex-Related Differences	Inconsistent Findings— Critical Issues	Knowledge Gaps	References
Systemic sclerosis	Pruritus affects ~40–65% of patients; often occurs early and may involve both affected and unaffected skin; associated with significant QoL impairment	Most studies (Canada, France): no significant sex differences in pruritus prevalence or severity, despite higher proportion of affected women	Use of self-reported measures; dichotomous itch assessment (yes/no); inability to capture fluctuations in severity; heterogeneous cohorts	Lack of validated multidimensional itch assessment; limited data on sex-related differences in pruritus severity and mechanisms	[108,159,162,163,164,171]
Morphea	Pruritus is frequently reported and associated with impaired HRQoL; often coexists with pain; itch is one of the most common symptoms reported by patients	No direct studies on sex differences; however, female sex is associated with worse QoL and higher symptom burden, including itch	Scarcity of epidemiological data; indirect evidence (QoL studies rather than itch-specific analyses); small cross-sectional cohorts	No studies specifically assessing sex differences in pruritus prevalence, severity, or mechanisms	[176,177,178,179]
Dermatomyositis	Pruritus reported in <50% to >90% of patients; often moderate-to-severe; correlates with cutaneous disease severity and reduced QoL	Single study: trend toward higher itch severity in females, but not statistically significant	Very limited number of studies; small sample size; lack of adjusted analyses; variability in itch measurement tools	Need for larger studies on sex differences; poor characterization of pathophysiological mechanisms and sensory profiles	[184,185,186,187,188,189]

Abbreviations: HRQoL: health-related quality of life; QoL: quality of life.

## 8. Conclusions

In conclusion, pruritus represents a common and clinically relevant symptom across a broad spectrum of dermatological diseases, including inflammatory, autoimmune, and connective tissue disorders. In inflammatory dermatoses, pruritus is highly prevalent across conditions such as AD, PSO, PN, and LSC, although its clinical prominence and impact may vary, ranging from a predominant feature in some diseases to a major contributing factor to disease burden in others. In comparison, pruritus in other disease groups, including organ-specific autoimmune, bullous, and connective tissue disorders, appears to be less consistently characterized and, in some cases, underrecognized, despite growing evidence of its clinical relevance and impact.

The pathogenesis of itch is multifactorial, reflecting complex interactions between immune activation, neural sensitization, and, in selected conditions, structural skin changes and fibrosis. Despite shared mechanisms, the clinical expression, intensity, and impact of pruritus vary considerably depending on the underlying disease.

Importantly, increasing evidence suggests that sex and gender may act as key modifiers of itch perception, clinical severity, and psychosocial burden. However, current data remain limited and heterogeneous, and sex-related differences in pruritus are still inconsistently reported across dermatological conditions. This highlights the importance of incorporating a gender medicine perspective into both clinical research and patient management.

Future studies should adopt standardized methodologies and include sex- and gender-specific analyses to better elucidate the biological and psychosocial determinants of pruritus. In addition, the identification and validation of pruritus-related biomarkers, including neuroimmune mediators such as IL-31, may improve disease monitoring, help identify patients at greater risk of severe itch, and support the development of personalized therapeutic strategies. However, further studies are needed to establish their clinical utility across different dermatological diseases. Furthermore, considering the substantial psychological impact associated with chronic pruritus and the bidirectional relationship between itch and mental health, multidisciplinary collaboration between dermatologists and mental health professionals may help optimize patient management and improve clinical outcomes. Together, these advances may facilitate a more personalized approach to the assessment and treatment of chronic itch in dermatological practice.

## Data Availability

No new data were created or analyzed in this study. Data sharing is not applicable to this article.

## Abbreviations

The following abbreviations are used in this manuscript:

AD	Atopic dermatitis
APC	Antigen presenting cell
BP	Bullous pemphigoid
CGRP	Calcitonin gene–related peptide
CP	Chronic pruritus
DH	Dermatitis herpetiformis
DM	Dermatomyositis
DRG	Dorsal root ganglia
GPCR	G protein coupled receptor
IFN-γ	Interferon gamma
IL	Interleukin
ILC	Innate lymphoid cell
LC	Langerhans cell
LS	Lichen sclerosus
LSC	Lichen simplex chronicus
Mrgpr	Mas related G-coupled protein receptor
NGF	Nerve growth factor
NRS	Numerical rating scale
PAR	Protease-activated receptor
PF	Pemphigus foliaceous
PSO	Psoriasis
PV	Pemphigus vulgaris
QoL	Quality of life
SP	Substance P
SSc	Systemic sclerosis
Th	Helper T cell
TRPA1	Transient receptor potential ankyrin 1
TRPV1	Transient receptor potential vanilloid 1
TSLP	Thymic stromal lymphopoietin
VAS	Visual analogue scale
